# The foundation of the rhubarb industry economy: investigating metabolites disparities of rhubarb between varieties and growing environments on the Tibetan plate

**DOI:** 10.3389/fphar.2024.1461523

**Published:** 2024-09-27

**Authors:** Jinpeng Zou, Wei Wu, Fang Wang, Kai Hou

**Affiliations:** ^1^ College of Management, Sichuan Agricultural University, Chengdu, Sichuan, China; ^2^ College of Agronomy, Sichuan Agricultural University, Chengdu, Sichuan, China

**Keywords:** industrial economic development, metabolites difference, rhubarb, UHPLC-QTOFMS, R.tanguticum, R. austral

## Abstract

**Objective:**

In Tibetan dietary and folk medicine practices, *Rheum austral* is commonly used as an alternative to *Rheum tanguticum*, and there is a prevailing belief that wild rhubarb should not be substituted by its cultivated counterpart. However, these traditions are not supported by scientific evidence, particularly concerning the differences in endogenous metabolites between cultivated and wild rhubarbs, as well as between officially recognized and non-official rhubarbs. These uncertainties have also been hindering the vertical integration development of the local rhubarb industry.

**Methods:**

In this study, ultra-high performance liquid chromatography-quadrupole time-of-flight mass spectrometry (UHPLC-QTOFMS) and biostatistical analysis were employed to systematically and comprehensively investigate the chemical constituents of rhubarbs from various sources, focusing on the differences in metabolic components between cultivated and wild rhubarbs.

**Results:**

The metabolic differences in rhubarb from various varieties and environments are pronounced. Among them, 39 differential metabolites were identified between cultivated *R. tanguticum* and wild *R. tanguticum*. cultivated *R. tanguticum* is rich in emodin, physcion, and rhapontigenin, whereas wild *R. tanguticum* exhibits a higher concentration of rhaponticin and is particularly abundant in anthraquinone compounds. Additionally, 33 differential metabolites distinguished wild *R. tanguticum* from wild *R. austral*, with *R. austral* being rich in stilbene derivatives and wild *R. tanguticum* predominantly containing coumarins. The correlations among these differential metabolites have also been further explored and presented.

**Conclusion:**

The metabolic disparities between cultivated and wild rhubarb varieties are substantial, with wild rhuabarb containing higher levels of effective components than its cultivated counterparts. However, wild varieties face issues with component instability and resource depletion, while cultivated varieties exhibit more stable effective components. Given these significant differences in metabolic components, it is essential to differentiate rhubarbs from various species and growing conditions to suit specific medicinal and dietary purposes effectively. This paper can lay a theoretical foundation for the vertical integration development of the rhubarb industry in Tibetan areas.

## 1 Introduction

Chinese Rhubarb (“Da-Huang” in Chinese) belongs to the genus Rheum L. in the family Polygonaceae. Their dried roots and rhizomes, the famed traditional Chinese medicine (TCM), have been entered the practitioners’ prescriptions for thousands of years in different nations, such as China, Korea and Japan (Bajracharya and Gupta, 2021; Wang and Wu, 2023). Some rhubarb species have also served locals for many years as fresh vegetable and horticulture plants (Mezeyová et al., 2021). At present, only three official rhubarb varieties, namely, Rheum palmatum L., Rheum tanguticum Maxim. ex Balf., and Rheum officinale Baill., enter the lists of China’s Pharmacopoeia (CPC, 2020). The Tibetan plateau in Sichuan is widely regarded as one of the suitable districts to cultivate rhubarb with excellent quality in China. Meanwhile, the rhubarb industry is also an important economic source for the Tibetan people. Expanding rhubarb from traditional cultivation to processing industry can help enhance its added value and strengthen the economy of ethnic regions. The competitive advantage and industrial foundation of Sichuan Tibetan rhubarb lie in its unique health ingredients and medical value. In addition to the diarrheagenic effect, rhubarbs cultivated in their suitable districts have plenty of other pharmacological effects, including anti-cancer activities, anti-bacterial activities, outstanding antioxidant activities, and antidiarrhoeal activities. Metabolomics of rhubarbs mainly include anthraquinones, anthraglycosides, anthrones, stilbenes, tannins, polyphenols, essential oil, and organic acids (Zhou et al., 2024). Anthraquinones and anthrones are commonly regarded as two effective components for the quality of official rhubarbs in China, which is the same as pharmacopoeia of some other countries (Li et al., 2024).

TCM herbs are the basic raw materials for the production of a large number of prescription drugs, China has relied on wild TCM resources for a long time. With the growth of the population, the demand for TCM continues to increase, which seriously threatens the sustainable development of TCM resources. Therefore, the development of herb cultivation has become the mainstream to supply the market’s demands for TCM. To achieve effective quality control of the whole production process of TCM and realize standardized production of TCM (Zhang et al., 2021), China’s authority has promulgated good agriculture practices (GAP) for cultivating TCM herbs. However, sometimes the insufficient effective ingredients in TCM herbs produced by GAP bases still affect the safety and efficacy of clinical medications (Miao et al., 2024).

The current general opinion agrees that the content of metabolites of medicinal plants depend on the environment and species (Li et al., 2020). Wild medicinal plants are generally considered to have better secondary metabolites than cultivated ones, so ethnopharmacology in Tibetan areas also generally believes that the quality of the wild rhubarb is superior to that of the cultivated one (Wei et al., 2007). Therefore, Tibetan enthusiasm for the wild official varieties remains undiminished. Meanwhile, ethnic Tibetan clinical nutritionists argue that some non-official varieties have same medicinal value and healthcare effects to the official wild rhubarb, according to the ethnobotanical statistics of the Fourth National Survey of Chinese Materia Medica (Huang and Zhang, 2017; Zou et al., 2023).

To cater to the market’s preference for wild rhubarb, some wild non-official rhubarbs, such as *Rheum austral*. D. Don., *Rheum webbianum*. Royle., are also widely exchanged in Tibetan areas. In the local herbal market, both official and non-official rhubarb varieties from different sources are not strictly differentiated and sold separately. Instead, they are mixed together in varying proportions, ultimately becoming a common source of vegetables and herbs for the Tibetan people (Zhao et al., 2023). The ongoing persistence of this situation raises an urgent concern regarding the rhubarb’s quality issue that requires immediate attention. However, the compositional differences of rhubarbs from different sources that are circulating in markets are still unclear, which threatens to the food safety and medicine safety of Tibetans. In order to ensure the safety of the clinical medication and traditional diet, while to promote the gradual vertical integration of the rhubarb industry in the local area, it is necessary to conduct a comprehensively differential metabolite analysis among the rhubarbs which are circulating in Tibetan areas.

There are certain methods to identify different rhubarb species, like morphological identification, microscopic identification, and chromatography. However, these methods are not objective (Sun et al., 2019). Metabolomics is an emerging discipline that can effectively integrate sample information and detect small molecule metabolites among samples more accurately and comprehensively. Furthermore, non-targeted metabolomics analyze all detectable metabolites in a sample without bias, obtaining relative quantitative differences for studying biological problem, compared to targeted metabolomics (Feng et al., 2022). Ultra high performance liquid tandem chromatography quadrupole time of flight mass spectrometry (UHPLC-QTOFMS) is widely used in life science, nutrition, plant, pharmacy, and other research fields because of its high sensitivity and precision, which uses more rich data to screen differentially expressed metabolites (DEMs) among samples (Yan et al., 2022). Previous metabolomics studies mostly focused on the experimental pharmacology of rhubarb (Jiang et al., 2021; Zhu et al., 2022). Recently, UHPLC-QTOFMS has played an important role in the identification of biomarkers in different rhubarb species, because the metabolites of plants are the basis of rhubarbs’ pharmacological experiments (Luo et al., 2021).

This study used UHPLC-QTOFMS to detect the differences in metabolites of rhubarb in different species and environments, as well as the correlation between active ingredients. This article not only provides a systematic understanding of the differences in metabolites and metabolic pathways of rhubarb from different sources, but also lays a theoretical foundation for the future food and pharmaceutical development of rhubarb.

## 2 Materials and methods

### 2.1 Sampling

The rhubarb samples for this study were sourced from Xiaojin County in the Garzê Tibetan Autonomous Prefecture, Sichuan Province. Group 1 (G1) was collected from local GAP bases, while Groups 2 (G2) and 3 (G3) were harvested from wild locales. Morphological identification conducted by Dr. Wu Wei and Dr. Hou Kai from Sichuan Agricultural University preliminarily classified G1 and G2 as *R. tanguticum* and G3 as *R. austral*. Each group comprised six samples, prepared for subsequent analysis (Zhang et al., 2022).

### 2.2 Metabolites extraction

100 mg of each sample powder was transferred to a 2 mL Eppendorf (EP) tube and extracted with 500 μL of a methanol (CAS: 67–56-1, LC-MS grade, CNW Technologies) and water mixture (v:v = 4:1, IS = 1,000:10). After vortexing for 30 s, the samples were homogenized at 45 Hz for 4 min. The mixture was then ultrasonicated for 1 h in an ice bath. Afterward, the samples were stored for 1 h at −20°C, followed by centrifugation at 12,000 rpm for 15 min at 4°C. The supernatant was then passed through a 0.22 μm filter into a fresh 2 mL tube for subsequent analysis (Farooq et al., 2020; Khan and Ali, 2015).

### 2.3 On-board testing

Under the control of the Shimadzu Nexera UHPLC LC-30A ultra-high performance liquid chromatograph, the analysis was conducted with the mobile phase parameters outlined in Table 1 (Yan et al., 2023). The chromatographic column used was a UPLC BEH C18 column (1.7 μm × 2.1 × 100 mm) purchased from Waters. The injection volume was set at 5 μL. The AB 5600 Triple TOF mass spectrometer, controlled by Analyst TF 1.7 software (AB Sciex), was capable of performing both primary and secondary mass spectrometry data acquisition via the IDA function. In each data collection cycle, molecular ions with the highest intensity and a count above 100 were selected for acquiring corresponding secondary mass spectrometry data. The bombardment energy was set at 40 eV, with a collision energy differential of 20 V, and 15 secondary spectra were captured every 50 m. The parameters for the ESI ion source were set as follows: atomization pressure at 55 Psi, auxiliary pressure at 55 Psi, curtain pressure at 35 Psi, temperature at 550°C, and spray voltage at 5500 V in positive ion mode (POS) or −4,000 V in negative ion mode (NEG).

**TABLE 1:** The mobile phase conditions of the liquid chromatography.

Time (min)	Velocity of flow (μL/min)	A% Water (0.1% formic acid)	B% Acetonitrile (0.1% formic acid)
0	400	95	5
3.5	400	85	15
6	400	70	30
6.5	400	70	30
12	400	30	70
12.5	400	30	70
18	400	0	100
22	400	0	100

*A: The mobile phase consists of 0.1% formic acid in the water; B: 0.1% formic acid in the acetonitrile.

### 2.4 Quality control (QC)

The detection of samples required considerable time, especially when dealing with large numbers. It was crucial to monitor the stability and signal of the instrument promptly. Timely identification and resolution of any abnormalities were essential to ensure the quality of the data ultimately collected. QC primarily includes process QC and data QC. Process quality relied on the stability of the instrument and the response of the internal standard (IS): l-2-chlorophenylalanine (CAS: 103,616–89-3, >98%, Shanghai Hengbai Biotechnology Co., Ltd). Data QC could be assessed based on the correlation of QC samples and the response stability of the IS in QC samples (Cao et al., 2020; Li et al., 2021).

### 2.5 Data processing and analysis

The original mass spectrum was imported into Progenisis QI software (ver. 2.4, Nonlinear Dynamics) for statistical analysis and correlation, the retention time correction, peak identification, peak extraction, peak integration, peak alignment and other pretreatment of original data carried out, and then the peaks were identified by using the self-built secondary mass spectrum database and the corresponding pyrolysis law matching method. Compound identification and reliable quantification were followed the methods described in previous literature (Alseekh et al., 2021).

**FIGURE 1:**
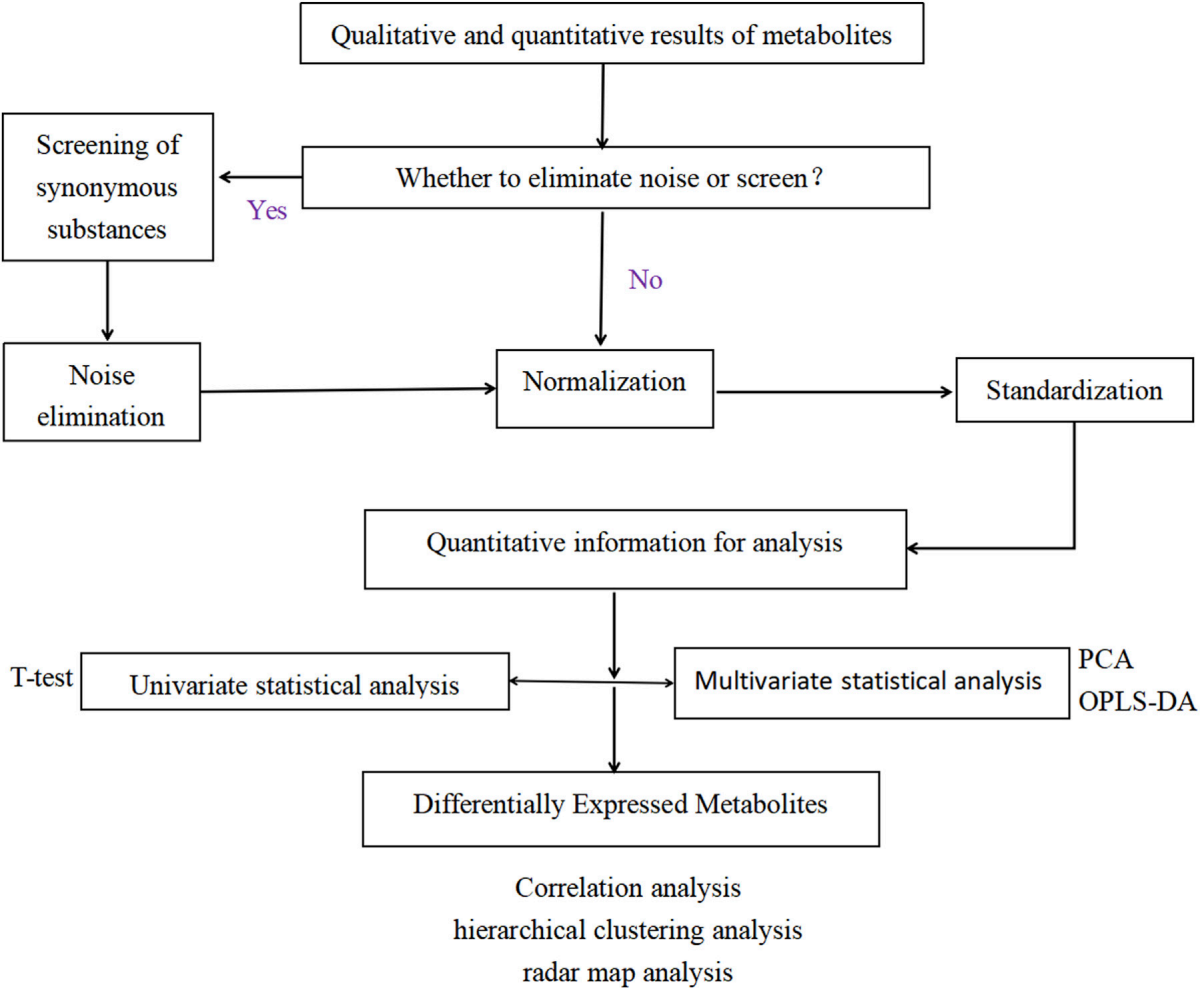
Data processing flow.

Then, the qualitative and quantitative results were analyzed by univariate statistics and multivariate analysis to screen DEMs, not only including student’s t-test, orthogonal projections to latent structures-discriminant analysis (OPLS-DA), Hierarchical cluster analysis (HHCA) of DEMs. LOG conversion and UV formatting was achieved through SIMCA software SIMCA software (V15.0.2, Sartorius Stedim Data Analytics AB, Umea, Sweden). Quality control was performed by checking ion intensity maps and producing alignments of all sample runs. The specific process of data processing is shown in Figure 1.

## 3 Result and analysis

### 3.1 Morphological identification

**FIGURE 2:**
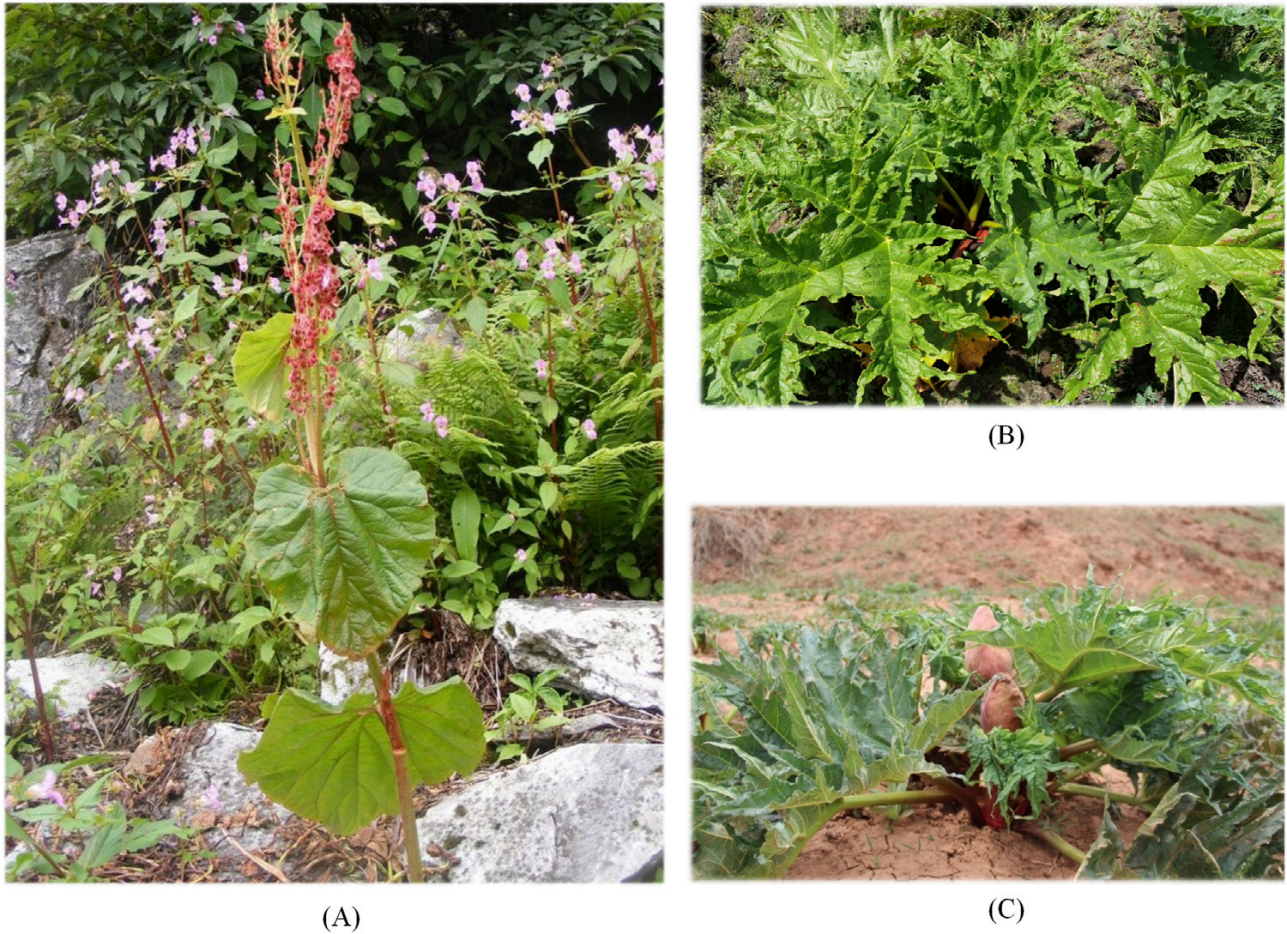
Morphological comparison of different sources of rhuabarb. **(A)** Wild *R. austral.* **(B)** Wild *R. tanguticum.* **(C)** Cultivated *R. tanguticum*.

From the comparison of the appearance and morphological characteristics of *R. austral* and *R. tanguticum*, *R. austral* had thick, erect, smooth, green or purplish stems. The leaves were bipinnate, alternate, rosette-shaped, with longer petioles. As shown in Figure 2A, the root of *R. austral* was stout, mostly cylindrical, dark yellow or brownish yellow in color, with obvious longitudinal lines, and uneven thickness. The difference between *R. austral* and *R. tanguticum* was mainly reflected in the characteristics of the leaf blade and inflorescence. The basal veins of the leaf blade of *R. austral* were usually 5, while those of *R. tanguticum* are 3; the inflorescence of *R. austral* was a large panicle, with tightly clustered branches and smaller flowers, while the inflorescence of *R. tanguticum* was a panicle, with more sparse branches and larger flowers. By comparing Figures 2B, C, wild *R. tanguticum* had larger, highly divided leaves of variable shapes and sizes, and thick, yellow, possibly rough-surfaced rhizomes, whereas cultivated *R. tanguticum* had smaller, less-divided leaves of standardized shapes and sizes, and thinner, smooth-surfaced rhizomes, which might be of different colors.

### 3.2 Pretreatment of original data

The original data included 5 QC samples and 18 experimental samples under negative or positive ion mode, from which 15,423 and 15,164 peaks are extracted under the two modes respectively. These peaks are preprocessed based on UHPLC-QTOF-MS, including removing noise from a single peak, filtering a single peak, simulating missing value recoding in the original data, and using internal standards for normalization. After pretreatment, 6,533 peaks and 5,174 peaks are reserved respectively under the two modes.

### 3.3 Orthogonal projections to latent structures discriminant analysis (OPLS-DA)

A typical statistical method used in metabolomics data processing is OPLS-DA. OPLS-DA can achieve a greater level of group separation, allowing for a better understanding of the factors involved for categorization (Lu et al., 2020).

Metabolome data can be considered as a multivariate dataset and can be displayed in a high-dimensional data spatial coordinate system, so the principal components analysis can provide a relatively low dimensional image (two-dimensional or three-dimensional), effectively using a small number of principal components to reduce the dimensions of data. However, the metabonomic data based on UHPLC-QTOF-MS has the characteristics of high dimension (more detected metabolites) and small samples (less detected samples). These variables include both differential variables related to categorical variables and a large number of non-differential variables that may be related to each other. As a result, the differential variables in principal components analysis will be scattered to more principal components, and better visualization and subsequent analysis cannot be carried out. Through OPLS-DA analysis, we can filter out the orthogonal variables which are not related to the classification variables, and analyze the non-orthogonal variables and the orthogonal variables respectively, so as to obtain more reliable metabolism information on the differences and correlation among groups (Trygg and Wold, 2002). SIMCA software (V15.0.2, Sartorius Stedim Data Analytics AB, Umea, Sweden) is used for LOG conversion and UV formatting of the data. Firstly, the first principal component is analyzed by OPLS-DA modeling. The quality of the model is verified by 7-fold cross validation. Then, the validity of the model was evaluated with R^2^Y (model’s interpretability to variable Y) and Q^2^ (model’s predictability) from the cross validation. Finally, through permutation test, different random Q^2^ were acquired by changing the order of the variables Y for many times (n = 200), and the validity of the model was further tested (Wiklund et al., 2008). OPLA-DA model parameters and the results of permutation test are shown in Supplementary Table S1, S2.

**FIGURE 3:**
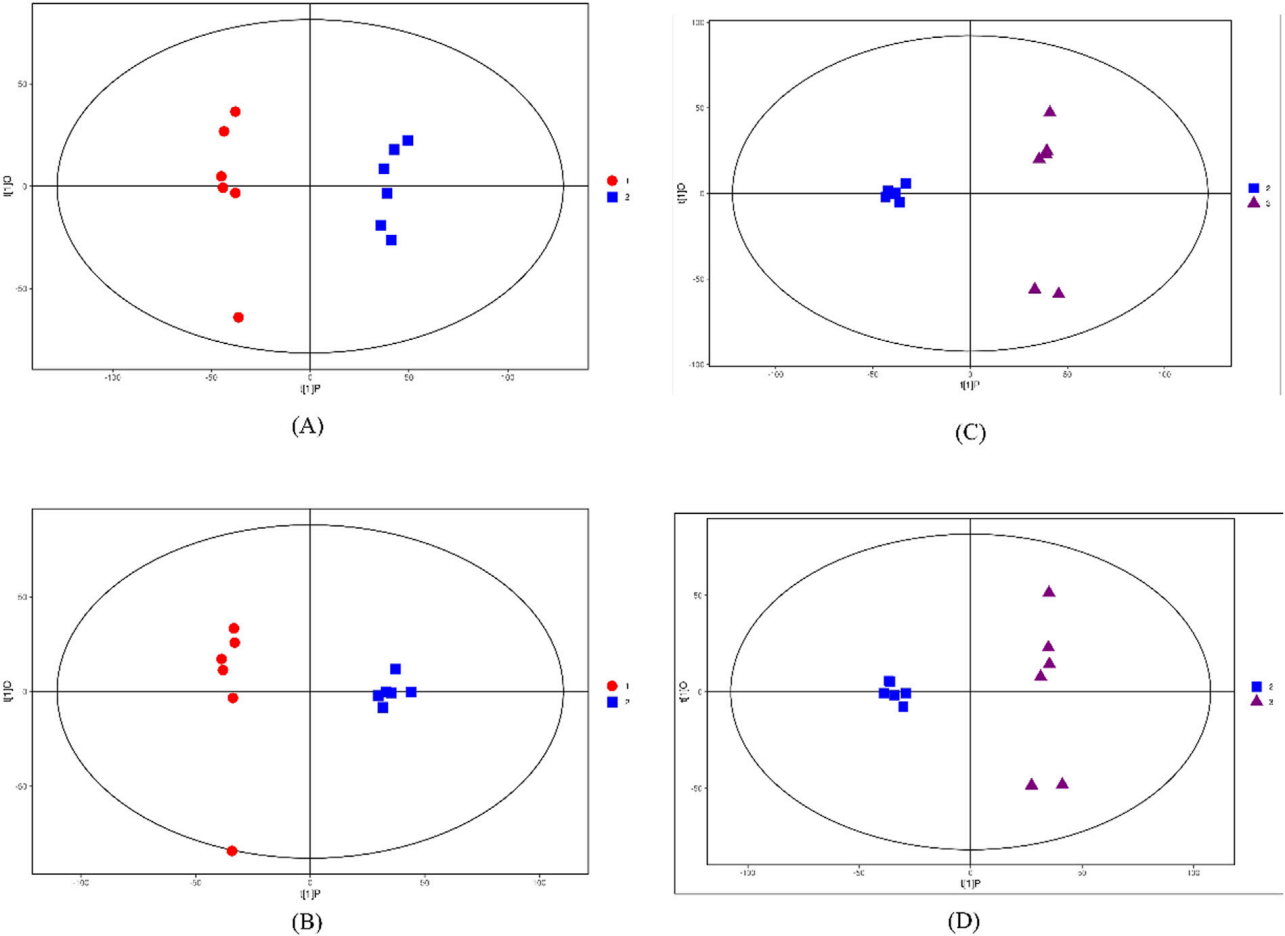
Score scatter plot of OPLS-DA model for G1 vs. G2 and G2 vs. G3 in the two modes. **(A)** Score scatter plot of OPLS-DA model for G1 vs. G2 in POS. **(B)** Score scatter plot of OPLS-DA model for G1 vs. G2 in NEG. **(C)** Score scatter plot of OPLS-DA model for G2 vs. G3 in POS. **(D)** Score scatter plot of OPLS-DA model for G2 vs. G3 in NEG. t [1]P represents the score of the predicted principal component of the first principal component, t [1]O represents the score of the orthogonal principal component.

It can be seen from Figure 3 that G1 vs. G3 or G2 vs. G3 has very significant differences in principal components in the two modes. At the same time, the samples are all within the 95% confidence interval (Hotel’s T-squared ellipse). In addition, the Q^2^ value of the permutation test random model is smaller than that of the original model; The intercept between the regression line of Q^2^ and the vertical axis is less than zero; Along with the gradual decrease of the retention of displacement, the proportion of Y variable of displacement increases, and the Q^2^ of the random model decreases, indicating that OPLS-DA has good robustness, and there is no over fitting phenomenon. Therefore, there are obvious differences in the principal components between the cultivated *R. tanguticum* and the wild *R. tanguticum*, wild *R. austral* and wild *R. tanguticum*. The distribution of the principal components of wild *R. tanguticum* is more dispersed which means that the results are stable and reliable.

### 3.4 Screening of differentially expressed metabolites (DEMs)

DEMs were screened according to following criteria in this paper: *P*-value of student's t-test is less than 0.05, and the variable importance in the project (VIP) of the first principal component in OPLS-DA model is more than 1. In POS, 1,455 and 567 differential metabolites are screened from G1 vs. G2 and G2 vs. G3, respectively. In NEG, 1,120 and 1,009 differential metabolites are screened from G1 vs. G3 and G2 vs. G3, respectively. See the supplementary excels for details (NEG-Differentially Expressed Metabolites. xlsx and POS-Differentially Expressed Metabolites. xlsx).

### 3.5 Expression comparison of differentially expressed metabolites (DEMs)

The obtained DEMs often have similarity or complementarity in biology or are positively/negatively regulated by the same metabolic pathway, showing similar or opposite expression characteristics among different experimental groups. HHCA of these characteristics can help us to classify the metabolites with the same characteristics into one group, and discover the changes of DEMs among the experimental groups. For each comparison, we calculate the Euclidean distance matrix for the quantitative value of the DEMs, to cluster the DEMs with the complete linkage method, and display the results with the heatmap. Moreover, the correlation coefficient of the quantitative value of different DEMs is calculated by the Pearson method and presented in the heatmap (Trygg and Wold, 2002).

All the DEMs with the matching score = 1 are included in the HHCA based on a self-built secondary mass spectrum database as well as effective components. In the heat map of this paper, the redder the color, the greater the relative expression of DEMs. For each comparison, the corresponding ratios are calculated based on the quantitative values of the DEMs, followed by 2 logarithmic transformation to make the radar chart.

#### 3.5.1 Cultivated *R. tanguticum* (G1) vs. wild *R. tanguticum* (G2)

**FIGURE 4:**
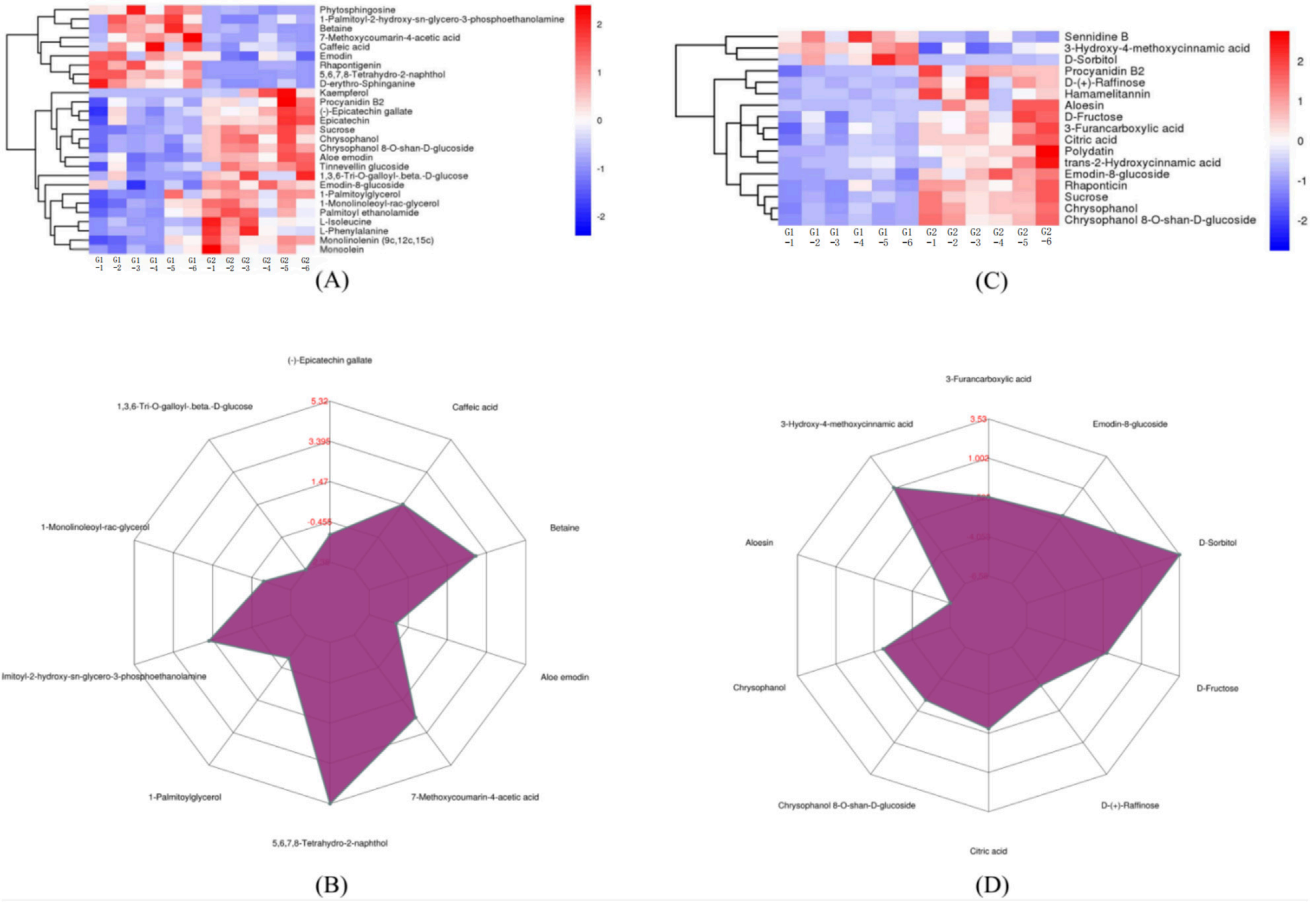
The expression comparison of the principal DEMs for G1 vs. G2. **(A)** Heatmap of HHCA of principal DEMs for G1 vs. G2 in POS. **(B)** Radar chart analysis for G1 vs. G2 in POS. **(C)** Heatmap of HHCA of principal DEMs for G1 vs. G2 in NEG **(D)** Radar chart analysis for G1 vs. G2 in NEG.

G1 and G2 groups exhibited 39 DEMs with a matching score of 1. As illustrated in Figures 4A, C, G1 is enriched in metabolites such as 1-palmitoyl-2-hydroxy-sn-glycero-3-phosphoethanolamine, 5,6,7,8-tetrahydro-2-naphthol, 7-methoxycoumarin-4-acetic acid, betaine, caffeic acid, D-erythro-Sphinganine, phytosphingosine, sennidine B, D-Sorbitol, and 3-hydroxy-4-methoxycinnamic acid. Notably, significant variances were observed in the levels of 5,6,7,8-tetrahydro-2-naphthol and D-sorbitol between the two groups, as shown in Figures 4B, D.

**FIGURE 5:**
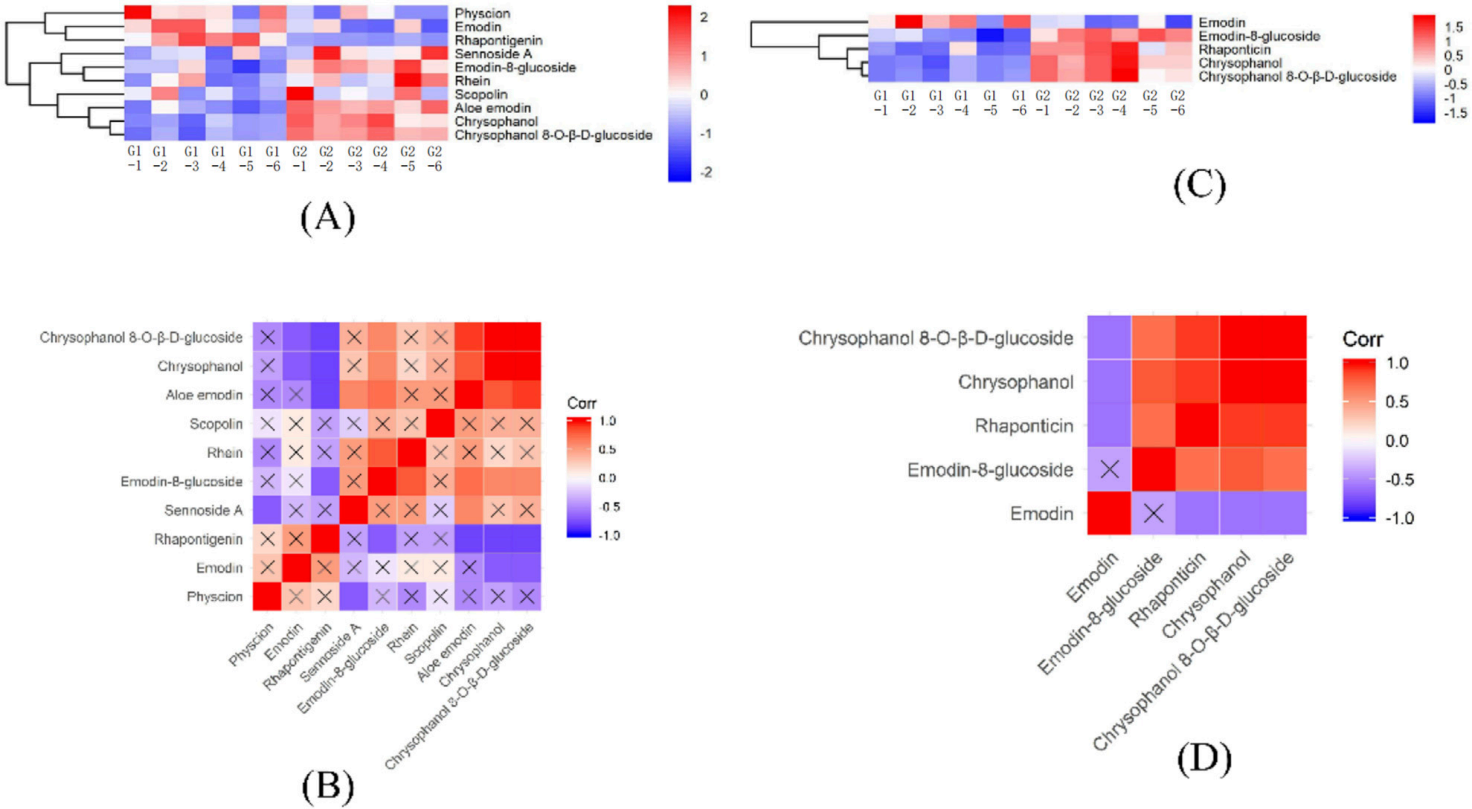
Heatmap of HHCA and correlation analysis of effective components in the DEMs for G1 vs. G2. **(A)** HHCA of effective component in the DEMs for G1 vs. G2 in POS. **(B)** Heatmap of correlation analysis of effective component in DEMs for G1 vs. G2 in POS. **(C)** HHCA of effective component in the DEMs for G1 vs. G2 in NEG. **(D)** Heatmap of correlation analysis of effective component in DEMs for G1 vs. G2 in NEG.

Regarding effective components, G1 predominantly contains emodin, physcion, and rhapontigenin, whereas G2 is characterized by a higher concentration of rhaponticin, as depicted in Figures 5A, C. Interaction analyses revealed that scopolin does not correlate with other effective compounds. emodin exhibits a specific negative correlation with rhaponticin, chrysophanol, and chrysophanol 8-O-β-D-glucoside, while physcion is negatively associated only with sennoside A. Notably, emodin does not relate to emodin-8-glucoside but shows negative interactions with chrysophanol and chrysophanol-8-O-β-D-glucoside. Conversely, rhein is positively correlated exclusively with emodin-8-glucoside, as detailed in Figures 5B, D.

#### 3.5.2 Wild *R. tanguticum* (G2) vs wild *R. austral* (G3)

**FIGURE 6:**
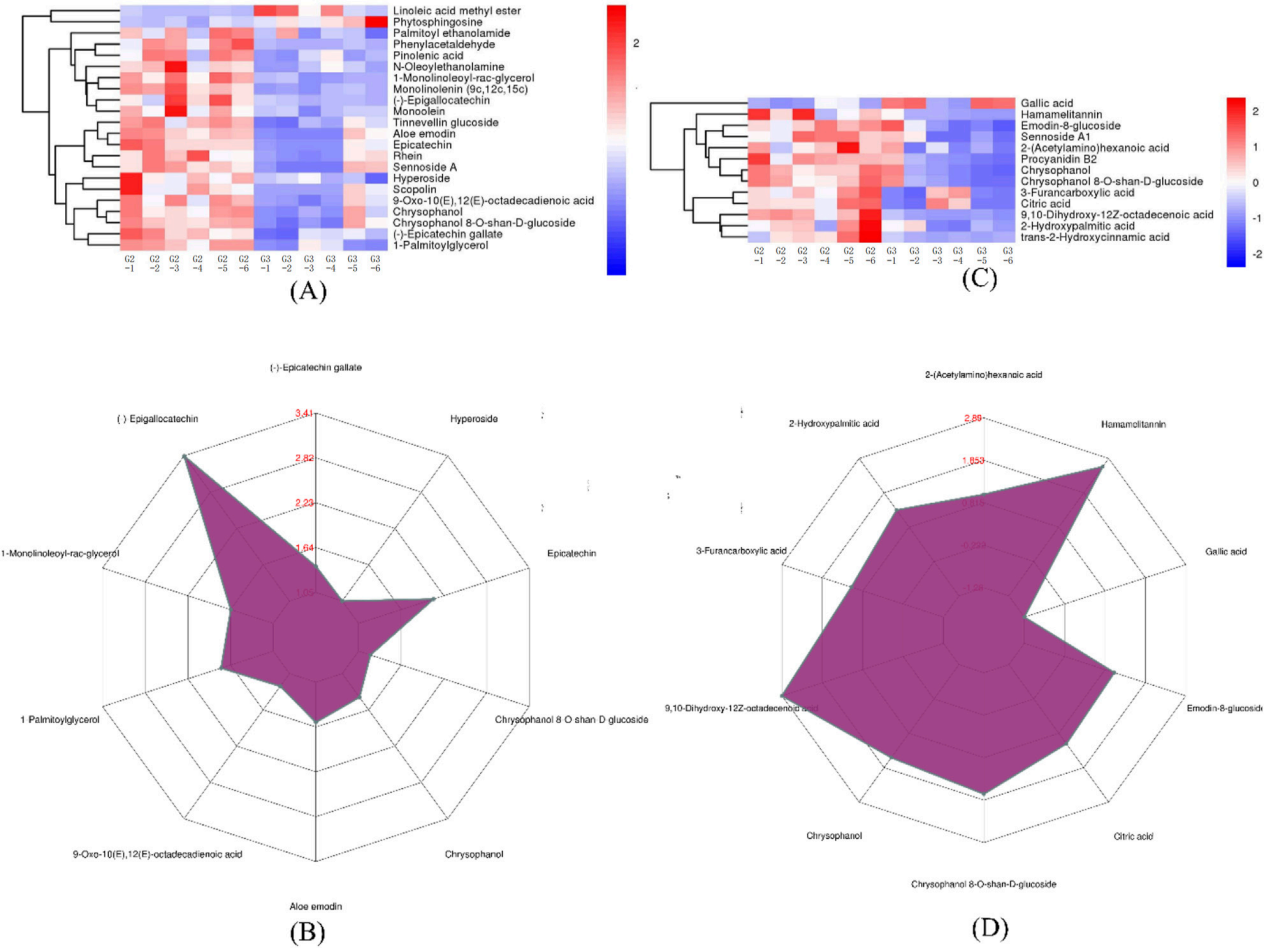
The expression comparison of the principal DEMs for G2 vs. G3. **(A)** Heatmap of HHCA of principal DEMs for G2 vs. G3 in POS. **(B)** Radar chart analysis for G2 vs. G3 in POS. **(C)** Heatmap of HHCA of principal DEMs for G2 vs. G3 in NEG **(D)** Radar chart analysis for G2 vs. G3 in NEG.

G2 and G3 groups exhibited 33 DEMs with a matching score of 1. According to Figures 6A, C, G2 showed higher concentrations of several metabolites including phenylacetaldehyde, pinolenic acid, N-oleoylethanolamine, 1-monolinoleoyl-rac-glycerol, monolinolenin (9c, 12c, 15c), (−)-epigallocatechin, epicatechin, monoolein, hamamelitannin, procyanidin B2, chrysophanol, chrysophanol 8-O-β-D-glucoside, and 9,10-dihydroxy-12Z-octadecenoic acid. Conversely, G3 was enriched with Linoleic acid methyl ester and phytosphingosine. Notably, G2 contains significantly higher levels of (−)-epigallocatechin, hamamelitannin, and 9,10-dihydroxy-12Z-octadecenoic acid, as depicted in Figures 6B, D.

**FIGURE 7:**
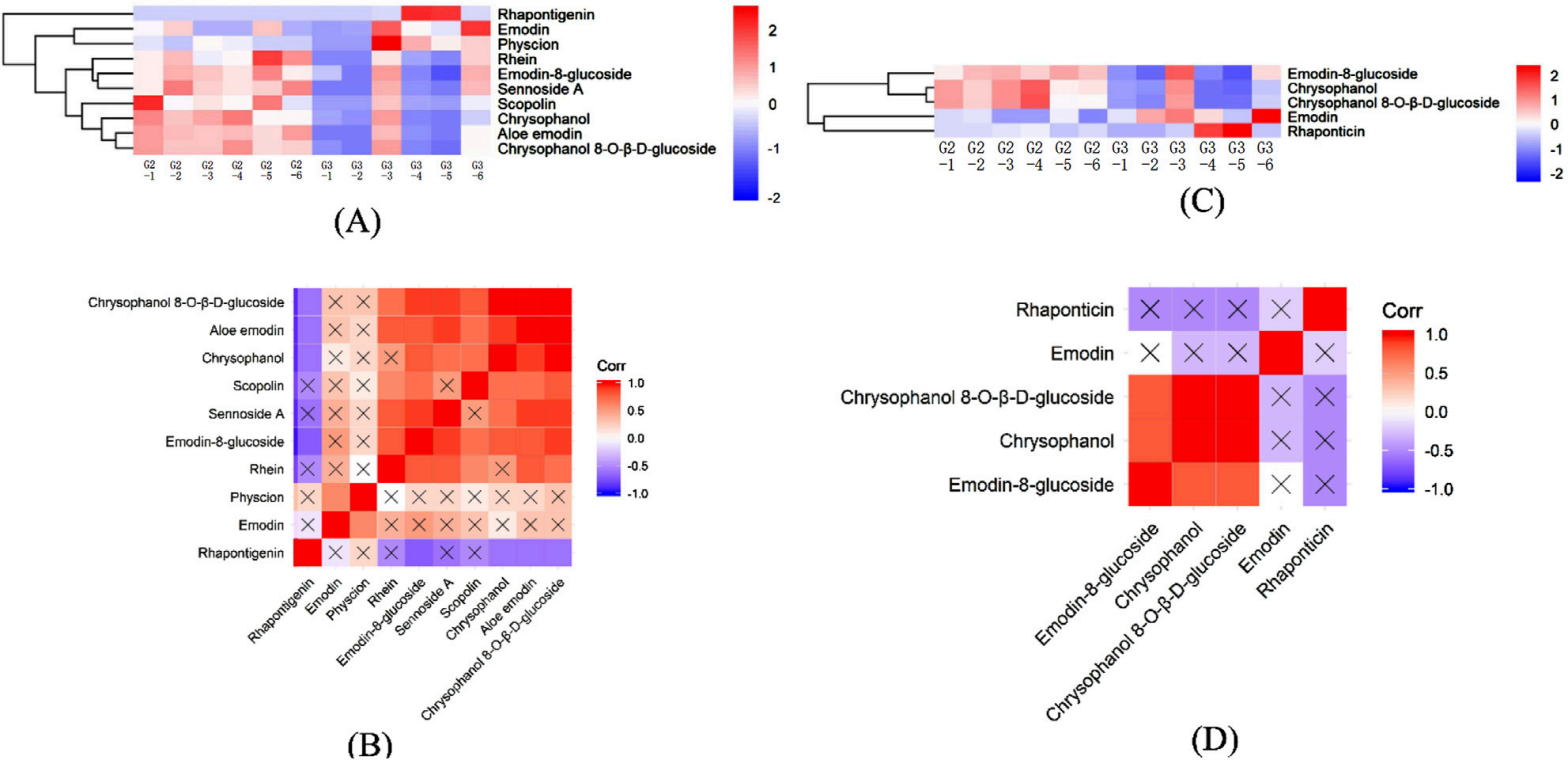
Heatmap of HHCA and correlation analysis of effective components in the DEMs for G2 vs. G3. **(A)** HHCA of effective component in the DEMs for G2 vs. G3 in POS. **(B)** Heatmap of correlation analysis of effective component in DEMs for G2 vs. G3 in POS. **(C)** HHCA of effective component in the DEMs for G2 vs. G3 in NEG. **(D)** Heatmap of correlation analysis of effective component in DEMs for G2 vs. G3 in NEG.

Effective component analysis highlighted that the metabolite profile of G3 (Figures 7A, C), particularly rhaponticin, emodin, and physcion, appeared more intense, suggesting a higher content. Similarly, the expression levels of scopolin and rhein in G2 were relatively higher, indicating that wild *R. austral* is rich in stilbene derivatives while wild *R. tanguticum* predominantly contains coumarins.

Further, Figures 7B, D revealed a strong positive correlation among chrysophanol 8-O-β-D-glucoside, aloe emodin, chrysophanol, scopolin, and emodin-8-glucoside, while these compounds displayed a negative correlation or no relationship with rhaponticin. Additionally, sennoside A showed no relationship with rhaponticin, emodin, physcion, or scopolin. Likewise, emodin was not related to emodin-8-glucoside, chrysophanol 8-O-β-D-glucoside, or chrysophanol.

### 3.6 Methodological verification

It was found that the retention time and signal strength of the total ion current peak of the QC sample overlapped well, indicating that the instrument had good stability (see Supplementary Figure S2). Meanwhile, the retention time and response strength of the extracted ion chromatography of the IS were stable, showing good data acquisition stability (Supplementary Figure S3). Similarly, the correlation of QC samples was close to 1 (Supplementary Figure S4), and at the same time, the response difference of the internal standard was less than 20% (Supplementary Table S2), all of which indicated that the system was stable and the data quality was reliable.

## 4 Discussion

Rhubarb is globally valued both as a medicinal agent and a dietary vegetable, known for its effectiveness in treating digestive issues and its potential protective effects against certain health conditions. Medically, rhubarb is used primarily for its laxative properties, aiding in constipation relief and promoting regular bowel movements. Additionally, it possesses anti-inflammatory and antioxidant properties, making it beneficial in managing inflammation and protecting against cellular damage. In the diet, rhubarb contributes essential dietary fiber and vitamins, supporting overall digestive health and wellness (Khattak et al., 2020). These health benefits have made rhubarb a subject of interest for its roles in both dietary safety and drug efficacy. In Tibetan regions, local residents consider both wild *R. austral* and wild *R. tanguticum* to possess equivalent medicinal values and health benefits. However, the quality of cultivated *R. tanguticum* is perceived to be inferior to that of its wild counterparts. However, this claim has yet to be fully substantiated through rigorous scientific scrutiny. Our investigation conducted a thorough analysis of the secondary metabolites present in these rhubarb variants, confirming that while the types of metabolites are consistent across samples, their concentrations exhibit substantial variation.

In this study, we identified significant differences in the major components among the three distinct sources of Rheum. The effective components of Rheum, such as scopolin, emodin, anthraquinones, coumarins, and rhaponticin, may be influenced by various growth environments, including climate, soil conditions, and altitude (Wink, 2003). These environmental factors potentially regulate secondary metabolic pathways in the plants, thereby affecting the accumulation of metabolites. Scopolin is a coumarin derivative, and coumarins generally play a role in plant defense mechanisms, particularly in response to biotic and abiotic stresses like ultraviolet radiation and pathogens (Gnonlonfin et al., 2012). The cultivated rheum (G1) and the wild types (G2 and G3) likely experience significant differences in light exposure, humidity, and soil composition, resulting in higher Scopolin content in G2 and G3. This observation aligns with reports in the literature on the accumulation mechanisms of coumarin compounds under various stress conditions (Yang et al., 2020).

Emodin and other anthraquinones are known for their significant bioactivities in rheum, including anti-inflammatory and antioxidant properties (Semwal et al., 2021). Studies suggest that the levels of anthraquinones are highly influenced by the plant’s genetic background and environmental conditions (Wang et al., 2013). The wild species (G2 and G3), being exposed to harsher natural environments, may accumulate more anthraquinones as a self-protection mechanism. Furthermore, anthraquinone accumulation may peak at specific stages of plant growth and development. The cultivated variety (G1), due to artificial selection and controlled growth environments, may exhibit different regulatory mechanisms affecting the accumulation of these compounds (Yang et al., 2020).

Genomic differences between the cultivated *R. tanguticum* (G1) and the *wild R. tanguticum* (G2) may also impact the synthesis of their metabolites. Through prolonged selective breeding, the cultivated species may have undergone changes in certain metabolic pathways, leading to differences in the content of specific metabolites. Rhaponticin, a significant stilbene derivative, is primarily found in the wild species (G2 and G3) (Scossa and Fernie, 2020). Research indicates that the synthesis of stilbenes is closely linked to plant stress resistance (Qayyum et al., 2022), and wild Rheum species may exhibit higher rhaponticin levels due to greater exposure to environmental stresses, while in cultivated species (G1), the reduced stress under artificial growing conditions may suppress Rhaponticin synthesis.

Therefore, the differences in the content of active compounds such as scopolin, emodin, anthraquinones, coumarins, and rhaponticin between G1, G2, and G3 can be attributed to variations in their growth environments, genetic backgrounds, and regulation of secondary metabolic pathways, as well as their differing physiological needs for plant defense. Future studies could further investigate these hypotheses through genetic analysis and metabolomic research to gain deeper insights into the metabolic differences between the various populations of rheum.

The effective components of rhubarbs sometimes have toxicity to human organs (Duan et al., 2018). Studies have reported that its effective components have chronic toxicity such as liver toxicity, nephrotoxicity, reproductive toxicity, and colon toxicity (Zhu et al., 2021). Therefore, long-term consumption of rhubarb has potential chronic toxicity. At the same time, this paper also found that wild *R. tanguticum* is richer in scopolin, which possesses both therapeutic effects and toxicity. Therefore, further toxicological research is needed to determine whether *R. tanguticum* can be included in the Tibetan diet, in order to establish a daily limit.

To enhance the clinical utility of rhubarb and reduce dietary risks, future research should focus on the precise selection and breeding of rhubarb varieties (Matvienko et al., 2022). Efforts should be directed towards developing therapeutic rhubarb varieties that can produce an increased array of effective components under ecologically sustainable cultivation practices, thereby enhancing their applicability in health and medical fields. Additionally, considering rhubarb’s traditional culinary use, it is crucial to develop edible varieties characterized by low toxicity, high health-promoting properties, and improved palatability. Such varieties would not only meet consumer demand for healthy food options but also strengthen rhubarb’s competitiveness in the food market. This integrated breeding strategy will directly respond to the global demand for safe, effective, and sustainable medicinal and food resources, facilitating the expansion of rhubarb applications into broader medical and culinary fields.

This study may also provide some new insights into the metabolic pathways of effective components in rhubarb. Scopolin, a phenolic coumarin, did not show significant correlations with other effective compounds in this study. This aligns with its unique pharmacological role, potentially acting independently of other metabolic networks. Scopolin has been recognized for its wide range of biological activities, such as antioxidant, hepatoprotective, and neuroprotective properties, as documented in various studies on its pharmacokinetics and pharmacodynamics (Gao et al., 2024). Emodin demonstrated negative correlations with rhaponticin, chrysophanol, and chrysophanol-8-O-β-D-glucoside, which may indicate distinct biosynthetic pathways or competitive interactions. Emodin’s role in ameliorating oxidative stress, particularly through the modulation of Notch-Nrf2 signaling, has been well-documented. This pathway is critical in regulating cell fate and antioxidative responses, as shown in research on emodin’s effects in fish models (Song et al., 2022). Additionally, the lack of interaction between emodin and its glucosylated form, emodin-8-glucoside, further emphasizes the complexity of their metabolic regulation. These findings support the idea that while certain anthraquinones, like emodin and chrysophanol, share common biosynthetic routes, they also exhibit distinct regulatory mechanisms depending on specific conditions. The positive correlations between compounds such as aloe emodin and chrysophanol-8-O-β-D-glucoside suggest shared roles in plant stress responses, particularly in regulating oxidative damage (Hu et al., 2022; Zhuang et al., 2020).

Based on the current research results, in order to further develop the rhubarb industry economy in the Tibet region, further rational planning can be carried out from the following aspects. Firstly, establish planting bases. It is recommended to establish standardized rhubarb planting bases to improve the quality and yield of rhubarb. Secondly, expand the industrial chain. It is recommended to further expand the industrial chain of rhubarb, develop deep-processing products of rhubarb, expand the market of rhubarb health products, and develop rhubarb cosmetic products. Thirdly, strengthen brand building: it is recommended to strengthen the publicity and promotion of rhubarb brands to improve brand awareness and reputation. Fourthly, study the active ingredients of rhubarb and their influencing factors: it is recommended to focus on the metabolic pathways of active ingredients and study the synthesis and accumulation mechanisms of these ingredients in different growth environments and genetic backgrounds. Fifthly, expand sales channels: it is recommended to strengthen cooperation with large-scale supermarkets, e-commerce platforms, etc., and broaden online and offline sales channels. Sixthly, vertical integration research and development: it is recommended to strengthen the technological research and development of the rhubarb industry and develop new rhubarb products and technologies.

## 5 Conclusion

In this study, the differential metabolic components among cultivated *R. tanguticum*, wild *R. tanguticum*, and wild *R. austral* were comprehensively analyzed using UHPLC-QTOFMS. Notably, 39 differential metabolites were identified between cultivated *R. tanguticum* and wild *R. tanguticum*, while 33 differential metabolites distinguished wild *R. tanguticum* from wild *R. austral*. These findings highlight significant variations in the content of principal and effective components across the groups. Both wild species contained rhaponticin; however, wild *R. austral* was particularly enriched in this compound, contrasting with wild *R. tanguticum*, which predominantly contained coumarins. Moreover, wild *R. tanguticum* exhibited a higher abundance of anthraquinones compared to its cultivated counterpart, underscoring the impact of natural *versus* cultivated growth conditions on the phytochemical profiles of rhubarb. Additionally, this study initiated a preliminary discussion on the correlation of metabolites of anthraquinone compounds, which will aid future analyses of the metabolic pathways of rhubarb’s byproducts.

These metabolic distinctions suggest differing pharmacological potentials and underline the importance of considering both species and cultivation practices in the utilization of rhubarb for therapeutic purposes. Given the dietary and medicinal use of rhubarb in Tibetan culture, it is crucial to differentiate based on differentially expressed metabolites to avoid potential chronic toxicity. Furthermore, there is a need to refine cultivation methods to enhance the content of effective components and improve the quality of cultivated rhubarb. The results of this study are robust and reliable, providing a valuable chemical composition reference for the quality control of botanicals. Meanwhile, this paper can also provide some ideas for the vertical integration development of the rhubarb industry economy.

## Data Availability

The original contributions presented in the study are included in the article/Supplementary Material, further inquiries can be directed to the corresponding authors.
