# Supplementary material for: The foundation of the rhubarb industry economy: investigating metabolites disparities of rhubarb between varieties and growing environments on the Tibetan plate
Source: Front Pharmacol. 2024 Sep 27;15:1461523. doi: 10.3389/fphar.2024.1461523 (PMC11467420; doi:10.3389/fphar.2024.1461523)
Supplement: Supplementary file 3 [file DataSheet1.docx]

Supplementary Material

**Supplementary Table 1.** PCA and OPLS-DA model parameter

| **Model** | **Type** | **A** | **N** | **R^2^X(cum)** | **R^2^Y(cum)** | **Q^2^(cum)** | **Title** | **Mode** |
| --- | --- | --- | --- | --- | --- | --- | --- | --- |
| Model 1 | principal components analysis | 4 | 23 | 0.579 |  |  | TOTAL with QC | POS |
| Model 2 | principal components analysis | 3 | 18 | 0.52 |  |  | TOTAL | POS |
| Model 3 | principal components analysis | 3 | 12 | 0.552 |  |  | 1-2 | POS |
| Model 4 | principal components analysis | 3 | 12 | 0.623 |  |  | 2-3 | POS |
| Model 5 | OPLS-DA | 1+1+0 | 12 | 0.427 | 0.99 | 0.89 | 1-2 | POS |
| Model 6 | OPLS-DA | 1+1+0 | 12 | 0.428 | 0.991 | 0.906 | 2-3 | POS |
| Model 1 | principal components analysis | 3 | 23 | 0.501 |  |  | TOTAL with QC | NEG |
| Model 2 | PCA | 3 | 18 | 0.536 |  |  | TOTAL | NEG |
| Model 3 | PCA | 3 | 12 | 0.534 |  |  | 1-2 | NEG |
| Model 4 | PCA | 2 | 12 | 0.526 |  |  | 2-3 | NEG |
| Model 5 | OPLS-DA | 1+1+0 | 12 | 0.46 | 0.989 | 0.885 | 1-2 | NEG |
| Model 6 | OPLS-DA | 1+1+0 | 12 | 0.433 | 0.987 | 0.867 | 2-3 | NEG |

*Model: Multivariate statistical analysis model number. Type: Model type, including principal components analysis, OPLS-DA or other types. A: The number of principal components. N: The number of observations of the model. R^2^X(cum): the cumulative explanation of the model for the variable X R^2^Y(cum) represents the cumulative explanation of the model for the variable Y. Q^2^(cum): predictability of the model. Title: The object corresponding to the model. Mode: Ionization mode.

**Supplementary Table 2.** Stability of IS in QC samples

| **IS** | **POS** | | | **NEG** | | |
| --- | --- | --- | --- | --- | --- | --- |
|  | m/z | R.T. | RSD_QC_ | m/z | R.T. | RSD_QC_ |
| **l-2-chlorophenylalanine** | 200.0469 | 2.44 | 3.67% | 198.0329 | 2.45 | 3.70% |

**
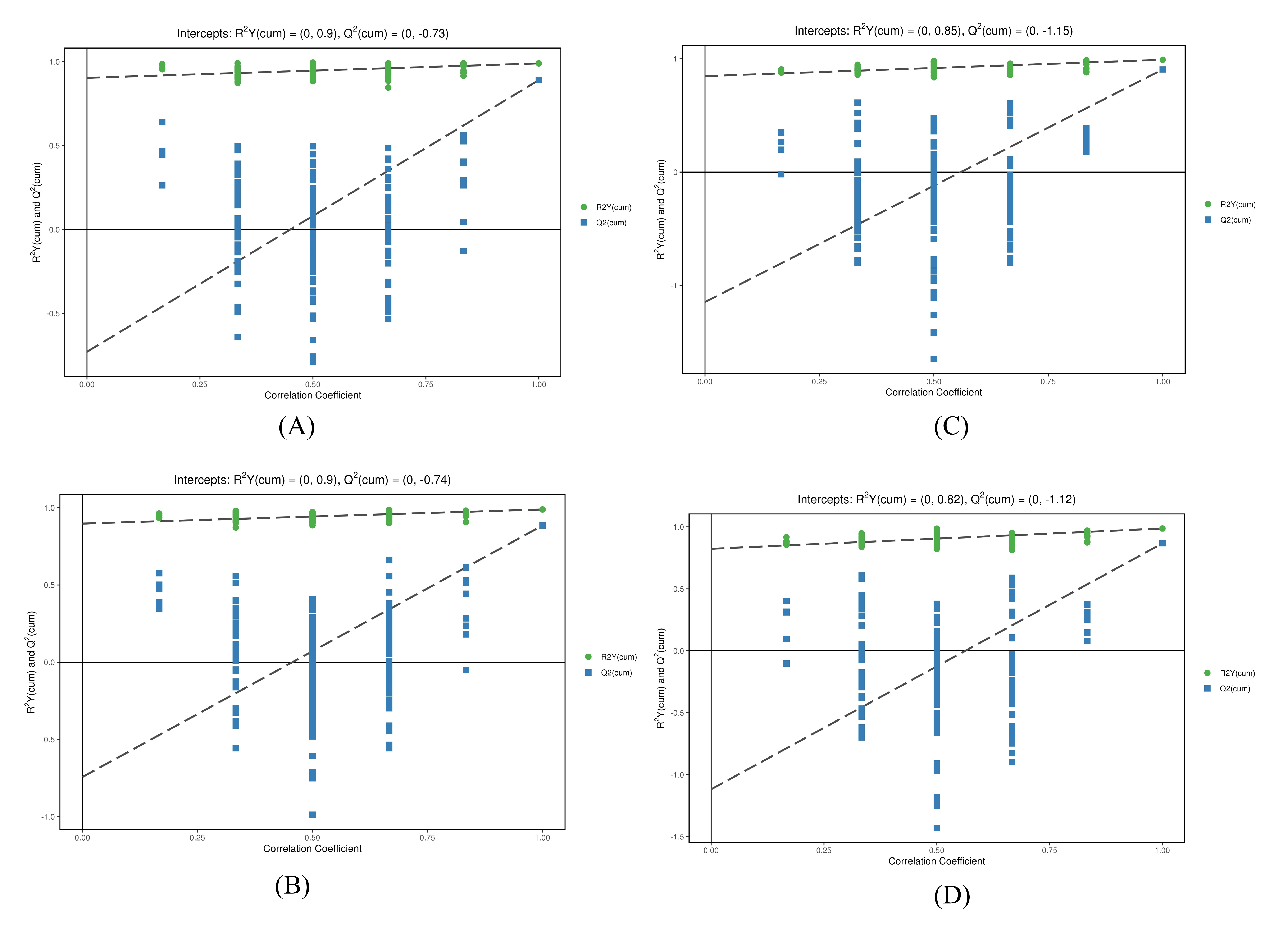
Supplementary Figure 1.** Permutation test of OPLS-DA model. (A) Permutation test of OPLS-DA model for G1 vs G2 in POS. (B) Permutation test of OPLS-DA model for G1 vs G2 in NEG (C) Permutation test of OPLS-DA model for G2 vs G3 in POS (D) Permutation test of OPLS-DA model for G2 vs G3 in NEG.


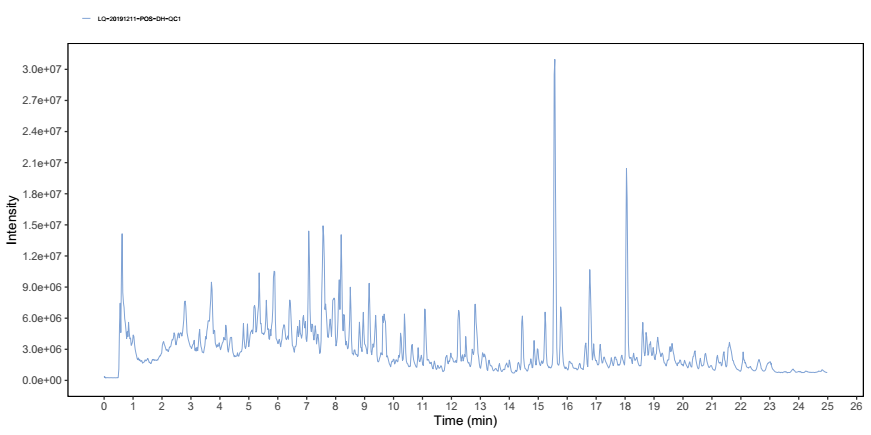

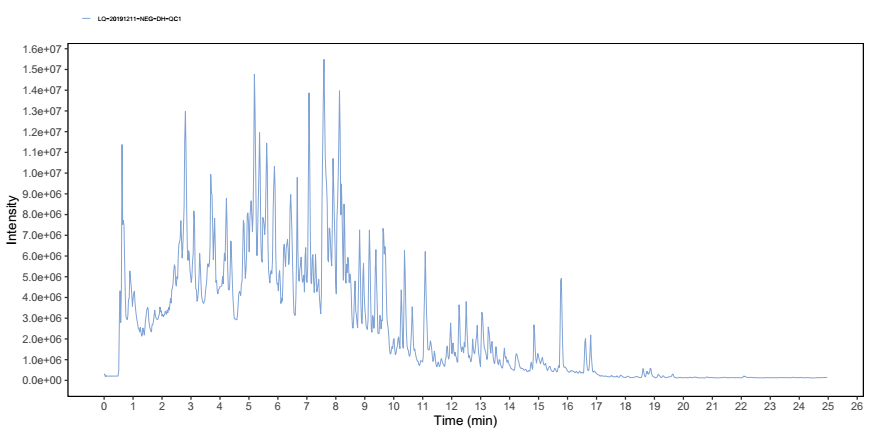
(A)

(B)

**Supplementary Figure 2.** TIC of QC. (A) TIC of QC in POS. (B) TIC of QC in NEG.


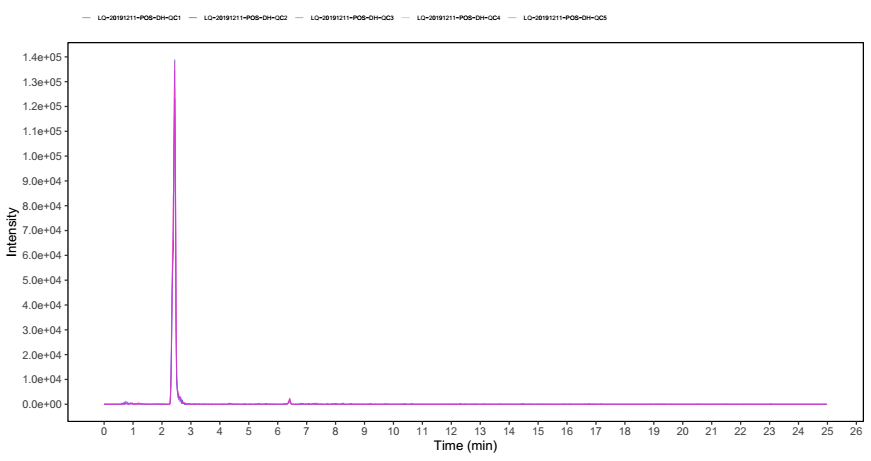
(A)


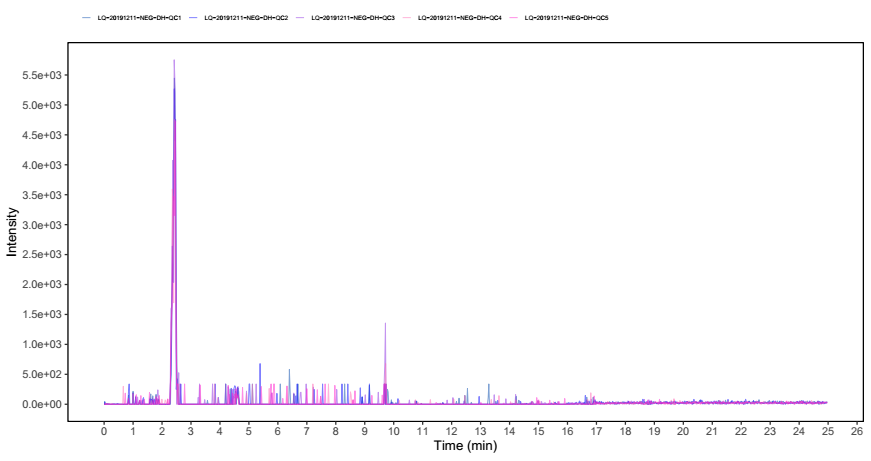


(B)

**Supplementary Figure 3.** Extract ion chromatography of IS. (A) Extract ion chromatography of IS in POS (B) Extract ion chromatography of IS in NEG.


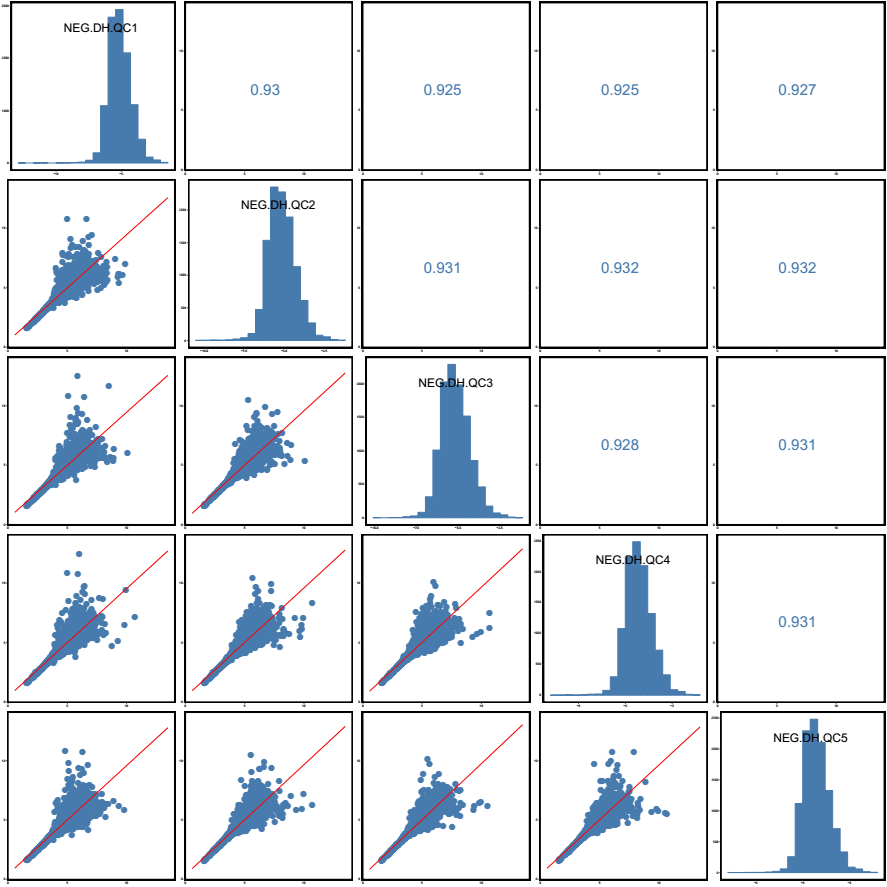


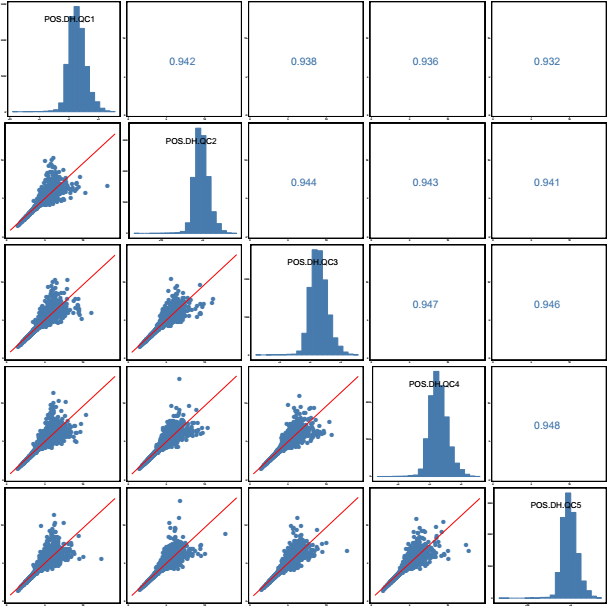


(A) (B)

**Supplementary Figure 4.** Correlation analysis of QC samples. (A) Correlation analysis of QC samples in POS. (B) Correlation analysis of QC samples in NEG.
